# A new Middle Triassic (Ladinian) trilophosaurid stem-archosaur from Germany increases diversity and temporal range of this clade

**DOI:** 10.1098/rsos.230083

**Published:** 2023-03-22

**Authors:** Hans-Dieter Sues, Rainer R. Schoch

**Affiliations:** ^1^ Department of Paleobiology, National Museum of Natural History, Smithsonian Institution, Washington, DC, USA; ^2^ Staatliches Museum für Naturkunde Stuttgart, Stuttgart, Germany; ^3^ Institut für Biologie, Universität Hohenheim, Stuttgart, Germany

**Keywords:** Reptilia, Triassic, Germany, Trilophosauridae, phylogeny

## Abstract

We report the first trilophosaurid stem-archosaur from Central Europe, *Rutiotomodon tytthos* gen. et sp. nov., from the Middle Triassic (Ladinian) Erfurt Formation of Baden-Württemberg (Germany). It is currently known from two jaw fragments with distinctive teeth. The labiolingually wide but mesiodistally narrow maxillary and dentary teeth each have a large labial cusp from which an occlusal ridge extends lingually to a small lingual cusp. A mesial and a distal cingulum extend between the labial and lingual cusps. The mesial and distal faces of the labial cusp each bear three prominent, lingually curved apicobasal ridges (arrises). A referred partial dentary has an edentulous, expanded symphysis similar to the mandibular ‘beak' in *Trilophosaurus buettneri*. A review of *Coelodontognathus ricovi*, from the Lower Triassic (Olenekian) of southwestern Russia, supports its referral to Trilophosauridae rather than Procolophonidae. Based on this reassessment and the new material from the Middle Triassic, the temporal range of trilophosaurids now spans nearly the entire Triassic Period, from the Olenekian to the Rhaetian. Trilophosaurids present craniodental features that indicate omnivory or herbivory with limited oral food processing. They were more diverse in terms of dental structure (and presumably diet) than previously assumed.

## 1. Introduction

Following the end-Permian extinction event, many new groups of terrestrial tetrapods, especially reptiles, originated or first diversified during the Triassic Period. Among them, various lineages of small- to medium-sized reptiles and synapsids evolved craniodental features for omnivory/herbivory and at least limited oral food processing [1]. This probably reflects, at least to some extent, the proliferation of new groups of ferns and gymnosperms during the post-extinction recovery of continental ecosystems [2]. During the late Permian, there were few known groups of small- to medium-sized herbivorous amniotes, almost all of which were dicynodont synapsids [3]. Most dicynodont lineages became extinct at the end of the Permian. During the Early and early Middle Triassic, procolophonid parareptiles [4,5] and rhynchosaurian stem-archosaurs [6] diversified alongside small- to medium-sized dicynodonts such as *Lystrosaurus* and *Myosaurus*, both from the *Lystrosaurus* Assemblage Zone of South Africa and Antarctica [3]. Non-mammalian therocephalian and cynodont synapsids with postcanine teeth suitable for omnivory or herbivory appeared in the late Early and early Middle Triassic [2,7]. In the late Middle and Late Triassic, dicynodonts were represented only by large-bodied kannemeyeriiforms [3], and hyperodapedontine rhynchosaurs and traversodontid cynodonts became the most common herbivores in Gondwana [8]. During or toward the end of the Late Triassic, most of these groups became extinct, and some rhynchocephalians [9,10] as well as small- to medium-sized synapsids including tritylodontid cynodonts [11] and haramiyidan mammaliaforms [12] evolved craniodental features for herbivory.

One clade of Triassic omnivorous/herbivorous reptiles that has received relatively little attention is Trilophosauridae, of which *Trilophosaurus buettneri*, from the Upper Triassic (Norian) of Arizona, New Mexico and Texas, is the best-known representative [13–15]. These stem-archosaurs are distinguished by the presence of labiolingually wide but mesiodistally short teeth in the maxilla and more posterior region of the dentary. Each tooth crown bears two or three labiolingually aligned cusps, which are usually linked by ridges. The upper and lower teeth interdigitated when the jaws were closed, and jaw motion was primarily orthal [16]. In *Trilophosaurus buettneri* and *T. jacobsi*, the premaxillae and the anteroposteriorly wide mandibular symphysis lack teeth and presumably bore a rhamphotheca in life. The cranium of *Trilophosaurus* is noteworthy for being dorsoventrally deep relative to its length, the presence of large upper temporal fenestrae separated by a tall, narrow sagittal crest, and dorsoventrally deep temporal bars without fenestrae or ventral emarginations.

*Trilophosaurus buettneri* was originally named on the basis of a fragment of a right maxilla (previously misidentified as a partial dentary) of a reptile with labiolingually wide but mesiodistally short teeth, each of which bears three labiolingually aligned cusps, from the Upper Triassic (Norian) Tecovas Formation of Crosby County, Texas [17,18]. It was referred to Procolophonidae. In 1940 and 1941, crews supported by the Works Progress Administration (an employment and infrastructure programme of the United States Government from 1935 to 1943) collected a nearly complete articulated skeleton and many other remains including jaws with teeth identical with those of Case's specimen from Norian-age strata of the Colorado City Formation in Howard County, Texas. This wealth of material allowed the first detailed description of *Trilophosaurus buettneri* [13]. The taxon was tentatively placed in Protorosauria, a now-abandoned group of what proved to be early-diverging archosauromorph reptiles.

Subsequently, two morphotypes of isolated reptilian jaws with *Trilophosaurus*-like teeth were reported from two localities exposing Late Triassic (Rhaetian) fissure fillings in southwest Britain [19,20]. *Tricuspisaurus thomasi* from South Wales and *Variodens inopinatus* from Somerset, England, both have transversely expanded tooth crowns with three labiolingually aligned cusps. *Tricuspisaurus thomasi* has an edentulous anterior portion of the mandible, whereas this is not the case in *Variodens inopinatus* [20]. Both are clearly very similar to *Trilophosaurus buettneri* [19]. The first phylogenetic review of the interrelationships of diapsid reptiles hypothesized archosauromorph affinities for Trilophosauridae [21], which have been recovered by numerous phylogenetic analyses since [22–25].

Since the work in the 1940s, many additional specimens of *Trilophosaurus* have been recovered from the Dockum Group of Texas and the Chinle Formation of Arizona and New Mexico [10,14,15]. Two additional species of this genus were recognized on the basis of diagnostic dental features: *T. jacobsi*, from the *Placerias* Quarry in the Norian-age lower Chinle Formation near St Johns in Arizona [14,26], and *T. dornorum*, from the stratigraphically younger Norian-age Sonsela Member of the Chinle Formation of Petrified Forest National Park in Arizona [27]. In unpublished theses, the skull of *Trilophosaurus buettneri* was described in more detail [28], and many features of its skeletal structure were clarified using microCT-scanning [29]. The enigmatic *Spinosuchus caseanus*, based only on a partial vertebral column from the Tecovas Formation of Crosby County, Texas, proved to be closely related to *Trilophosaurus* [30]. It is especially characterized by elongate, rod-like neural spines on the cervical, transitional and dorsal vertebrae. It is possibly a subjective senior synonym of *Trilophosaurus jacobsi* based on several co-occurrences of skeletal remains of both nominal taxa [23]. A fourth species, *Trilophosaurus phasmalophos*, from the Sonsela Member of the Chinle Formation in Petrified Forest National Park in Arizona, is distinguished by teeth with two rather than three labiolingually aligned cusps [31].

*Teraterpeton hrynewichorum* is known from a fairly complete skull and partial postcranial skeleton as well as other skeletal remains from the Upper Triassic (Carnian) Evangeline Member of the Wolfville Formation of Nova Scotia, Canada [25,32]. Its skull differs considerably from those of *Trilophosaurus buettneri* and *T*. *jacobsi*, particularly in the elongation of the snout (and external narial fenestra) and the structure of the teeth. In addition, an isolated tricuspid tooth crown from the Middle Triassic Economy Member of the Wolfville Formation was identified as an indeterminate trilophosaurid [33].

**Figure 1.**
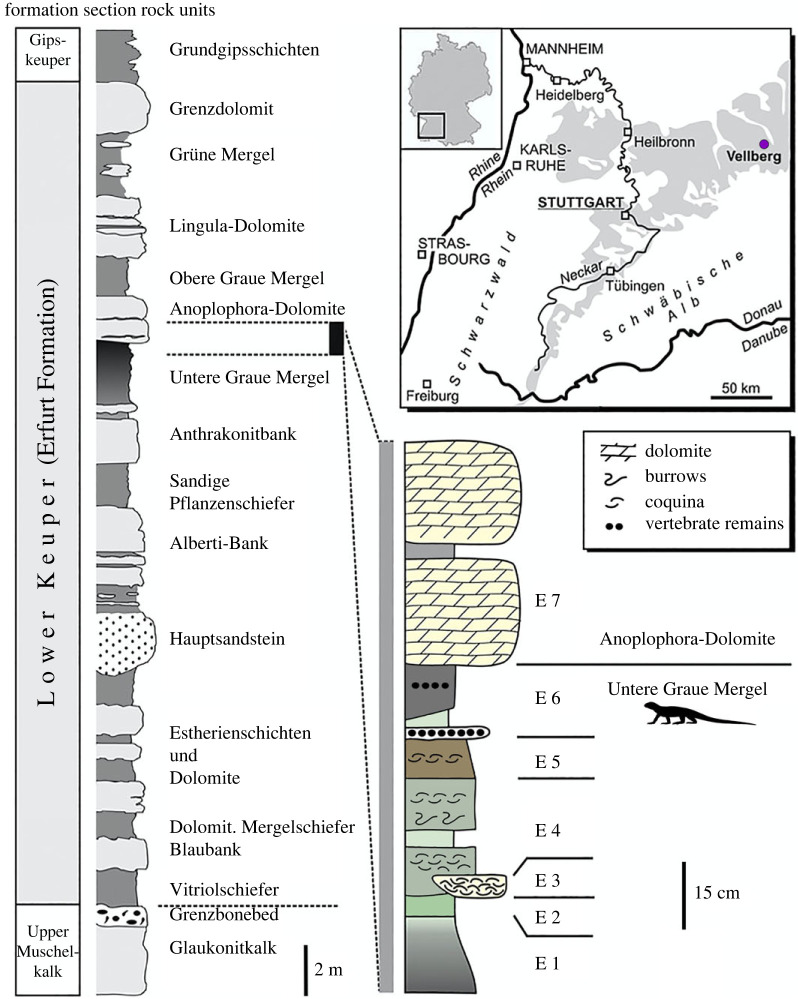
Stratigraphic succession of the Erfurt Formation (Lower Keuper) in southwestern Germany and measured section in the Schumann quarry near Eschenau, municipality of Vellberg, Baden-Württemberg (Germany). The silhouette of a trilophosaur (based on the skeletal reconstruction in [13]) marks the fossil-bearing stratum. The inset map shows the distribution of Keuper strata (in grey) in southwestern Germany and the location of Vellberg.

Since 1980, many skeletal remains of both aquatic and terrestrial tetrapods have been recovered from strata of the Erfurt Formation (also known as Lower Keuper or Lettenkeuper) exposed in an active limestone quarry (Schumann quarry) near Eschenau, in the municipality of Vellberg in Baden-Württemberg, Germany (figure 1) [34]. The Erfurt Formation is a mixed siliciclastic-carbonate sequence, which overlies the marine strata of the Muschelkalk Group and extends across the Central European Basin, from Switzerland in the south to the Baltic Sea in the north. It is dated as late Middle Triassic (Ladinian: Longobardian). In northern Baden-Württemberg, strata of the Erfurt Formation attain a thickness between 20 and 25 m. In the Schumann quarry, the most fossiliferous horizon is a layer of dark grey claystones (Untere Graue Mergel; unit 6 [34]). It attains a thickness of 5–12 cm at this locality and was apparently deposited in a small but fairly deep lake with periodically stagnant bottom waters. Streams washed plant debris and remains of land- and stream-dwelling tetrapods into the lake. Furthermore, desiccation cracks attest to repeated dry periods that probably introduced terrestrial animals into the depositional environment [34].

Among the wealth of tetrapod fossils from the Vellberg fossil *Lagerstätte*, we recently identified a right maxilla and a partial dentary, which both present features resembling those in *Trilophosaurus* spp. but differ considerably in the structure of the teeth. It is the purpose of this paper to describe these specimens and assess their phylogenetic relationships. We also took this opportunity to consider the phylogenetic positions of some other small-sized Triassic reptiles that have labiolingually wide but mesiodistally short teeth.

*Institutional abbreviations*. PIN, Borissiak Paleontological Institute, Russian Academy of Sciences, Moscow, Russia; SGU, former collection of Saratov Chernyshevsky State University, Saratov, Russia (now housed at PIN); SMNS, Staatliches Museum für Naturkunde Stuttgart, Stuttgart, Germany; TxVP, Texas Vertebrate Paleontology Collection, The University of Texas at Austin, Austin, Texas, USA; USNM, National Museum of Natural History, Smithsonian Institution, Washington, DC, USA.

## 2. Material and methods

For the assessment of the phylogenetic position of the new taxon, we scored its features into the character-taxon matrix published by Chambli-Trowell *et al*. [20], which is a modified version of that used by Kligman *et al*. [10], itself an iteration of the matrix compiled by Pritchard *et al*. [35]. The modified matrix used here (see electronic supplementary material) consists of 36 taxa and 231 parsimony-informative characters. It was analysed using the heuristic search option in PAUP* 4.0a [36]. Following [20], 10 characters—1, 4, 9, 10, 19, 31, 47, 62, 181 and 189—were treated as ordered. We modified the scoring for character 85 because none of the known trilophosaurids has interdental plates. In addition to the new taxon from Germany, we also added *Coelodontognathus ricovi* from the Lower Triassic (Olenekian) Lipovskaya Svita of Russia to test an earlier suggestion [4] that this species represents a trilophosaurid rather than a procolophonid.

MicroCT-scanning did not provide sufficiently high resolution to review structural details of the maxilla, presumably because the bone contains pyrite crystals that affect scan resolution.

Systematic Palaeontology

Diapsida Osborn, 1903 [37]

Archosauromorpha Huene, 1946 [38] *sensu* Dilkes, 1986 [22]

Allokotosauria Nesbitt, Flynn, Pritchard, Parrish, Ranivoharimanana, and Wyss, 2015 [23]

Trilophosauridae Gregory, 1945 [13]

*Rutiotomodon tytthos*, gen. et sp. nov.

*Nomenclatural acts*. This publication and its nomenclatural acts are registered at ZooBank. The publication is registered under LSID urn:lsid:zoobank.org:pub:017BC0F8-634D-42B0-9CCD-F38F16D61818, the new genus *Rutiotomodon* under LSID urn:lsid:zoobank.org:act:644E39B9-AB8B-4444-B7E2-CB8F63E282B4, and the species *Rutiotomodon tytthos* under LSID urn:lsid:zoobank.org:act:6FB1605-ADF9-489A-9F3F-77232BF63AFF.

**Figure 2.**
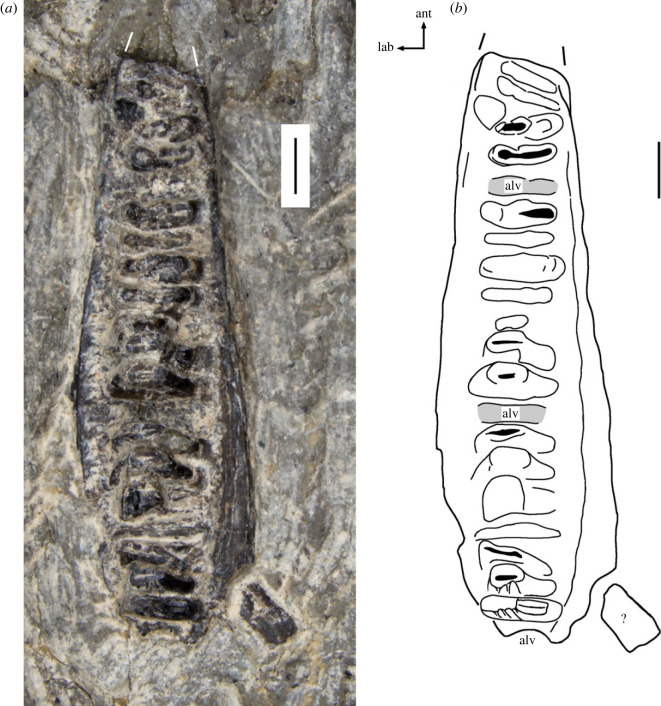
Right maxilla of *Rutiotomodon tytthos* (SMNS 97028, holotype). (*a*) right maxilla in occlusal view. (*b*) Outline drawing in the same view. Abbreviations: alv, empty alveolus; ant, anterior; lab, labial. The question mark marks an unidentified fragment. Scale bars each equal 1 mm.

**Figure 3.**
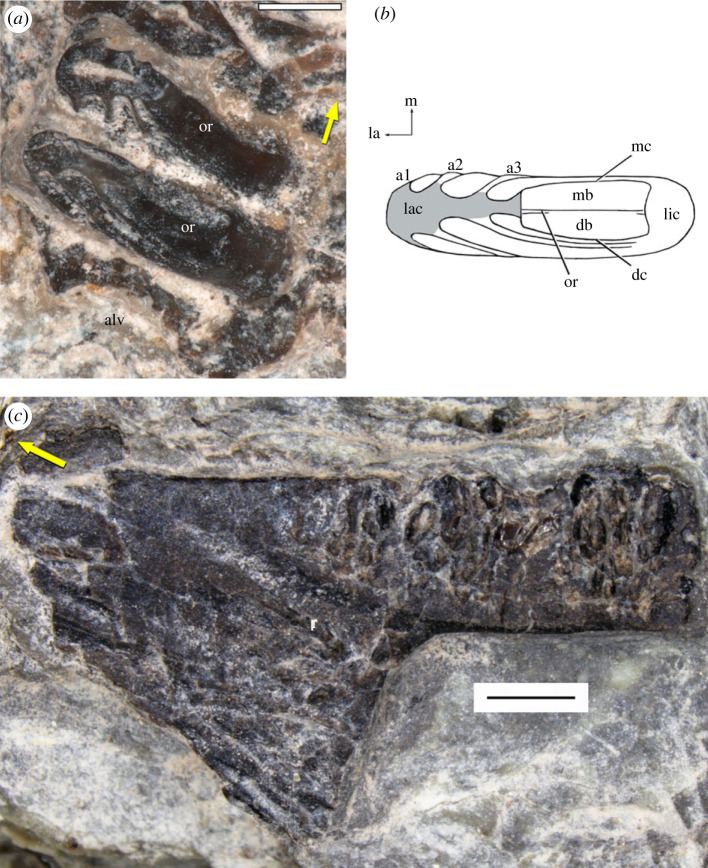
(*a*) Close-up of the posterior two maxillary tooth crowns and part of an empty alveolus distal to them in slightly oblique occlusal view. (*b*) Simplified rendering of the posteriormost tooth crown (position 20) in occlusal view. Shaded area denotes broken surface of labial cusp. (*c*) Right dentary fragment of *Rutiotomodon tytthos* (SMNS 97029) in occlusal view. Abbreviations: a1–3, apicobasal ridges (arrises) 1–3 on labial cusp; alv, empty alveolus; db, distal basin; dc, distal cingulum; la, labial; lac, labial cusp; lic, lingual cusp; m, mesial; mb, mesial basin; mc, mesial cingulum; or, occlusal ridge; r, ridge on lingual surface of symphysis. Yellow arrows point anteriorly. Scale bars equal 400 µm (*a*) and 1 mm (*c*).

*Holotype*. SMNS 97028, a nearly complete right maxilla with teeth, exposed in occlusal view (figures 2 and 3*a,b*).

*Referred Specimen*. SMNS 97029, crushed dentary fragment, preserving part of the symphysis and part of the right tooth-bearing ramus in occlusal view (figure 3*c*). Given the poor state of preservation of the teeth, its referral to *Rutiotomodon tytthos* is based on the large labial cusp of the posteriormost preserved tooth, which bears three distinct apicobasal ridges on its distal surface.

*Etymology*. Derived from Greek *rhytis*, wrinkle, fold, *tomō*, to cut, and *odon*, tooth, in reference to the prominent apicobasal ridges (arrises) on the labial cusp on the maxillary and dentary teeth and the inferred function of the tooth crowns. The species name is the Greek word *tytthos*, small.

*Locality and stratigraphy*. Schumann limestone quarry, near Eschenau in the municipality of Vellberg, Baden-Württemberg (Germany). Geographical coordinates: 49°4′33.71′′ N, 9°54′7.72′′ E. Top of the Untere Graue Mergel (lower grey marls; unit E6 [34]; figure 1), Erfurt Formation (Lower Keuper Subgroup of Keuper Group); age: late Middle Triassic (Ladinian: Longobardian).

*Diagnosis*. Maxillary and dentary teeth with large labial and small lingual cusp linked by labiolingually extending occlusal ridge and by mesial and distal cingula. Mesial and distal surfaces of labial cusp each bearing three prominent apicobasal ridges (arrises). Mandibular symphysis anteroposteriorly wide, edentulous, with thin lateral and anterior edges.

*Ontogenetic status*. The ontogenetic status of the specimens cannot be assessed because no remains suitable for skeletochronological analysis have been recovered to date.

## 3. Description

### 3.1. Maxilla

The nearly complete right maxilla SMNS 97028 is exposed in occlusal (=ventral) view (figure 2). It lacks its anterior end, which left a partial impression in the matrix. The length of the preserved portion of the bone is about 10 mm. The lateral margin of the maxilla is slightly convex anteroposteriorly, and its medial margin is somewhat thickened and becomes wider transversely more posteriorly. The teeth are labiolingually wide and mesiodistally short. There are 20 closely spaced teeth and alveoli in various stages of preservation. The anterior four teeth are damaged and separated from the remainder of the tooth row by a gap in the fifth tooth position. Teeth 6–12 are damaged, with the 12th being the most completely preserved. Separated by a gap in tooth position 13, the tooth positions 14–20 are occupied by teeth in various stages of completeness. Teeth 19 and especially 20 have nearly complete crowns, on which only the apices of the labial cusps have been lost. There appears to be the mesial margin of an empty alveolus for an additional tooth at the posterior end of the maxillary tooth row, suggesting the presence of a more rounded, smaller tooth, perhaps similar to the condition in some specimens of *Trilophosaurus buettneri* [15]. As in other trilophosaurids, the gaps in the tooth row are the result of tooth replacement rather than artefacts of preparation or preservation [16]. The maxillary teeth increase only slightly in labiolingual width posteriorly. Their number is much higher than in *Trilophosaurus buettneri*, which has up to 15 maxillary teeth/tooth positions [13], and *T*. *jacobsi*, which has 11 [14].

The structure of the maxillary teeth of SMNS 97028 differs from those of all previously described trilophosaurids. Teeth 19 and 20 have the best preserved crowns (figure 3*a,b*). The tooth crown is roughly elliptical in occlusal view, with its labiolingual width being much greater than its mesiodistal length. It bears a large labial cusp (which lacks its apex on all teeth due to breakage) from which an occlusal ridge descends on the mesiodistally concave lingual face of the cusp and then curves lingually to a lingual cusp. On the teeth anterior to position 20, this ridge is apicobasally taller and extends more steeply toward the lingual end of the crown. On tooth 20, the mesial and a distal cingulum link the labial and lingual cusp and enclose a basin, which is traversed by the occlusal ridge. A faint distal cingulum is present on tooth 19. The mesial and distal surfaces of the labial cusp each are fluted with three apicobasal ridges (arrises), which are triangular in cross-section. On the mesial and distal surfaces of the labial cusp of the posterior teeth, the arrises extend increasingly more lingually from the labial ridge to the lingual one.

The maxillary teeth are rooted in rather shallow alveoli and surrounded by finely pitted bone. This mode of tooth implantation has most recently been termed ‘protothecodont' [20].

### 3.2. Dentary

The incomplete right dentary SMNS 97029 is exposed in occlusal (=dorsal) view, preserving much of the anteroposteriorly wide symphysis and the anterior portion of the right mandibular ramus (figure 3*c*). Its greatest length (measured along the labial margin of the ramus fragment) is 8 mm. It is possible that part of the splenial is present along the lingual surface of the tooth-bearing ramus but its presence or absence could not be ascertained. The teeth are badly damaged and little detail of the tooth crowns is evident. There appear to be 11 teeth and at least one empty alveolus mesial to the first tooth. Furthermore, an empty socket separates the posterior three teeth from the remainder of the tooth row. The tooth crown of the posteriormost tooth row shows traces of apicobasal ridges on its distal surface. The teeth sit in shallow alveoli. The extensive mandibular symphysis is separated from the tooth row by a lingual ridge, which continues the lingual margin of the dentary tooth row anteriorly. The symphysis lacks teeth, and its right lateral margin curves anteromedially. It has been flattened during fossilization, and thus it cannot be ascertained whether it formed a transversely concave ‘spout' as in *Trilophosaurus buettneri* [13,15]. A short ridge extends anteriorly on the lingual surface of the symphyseal region parallel to the lingual ridge of the mandibular ramus. Owing to the crushing, the presence or the absence of a sutural contact between the dentaries cannot be ascertained.

## 4. Phylogenetic relationships

*Rutiotomodon tytthos* resembles Trilophosauridae more derived than *Teraterpeton hrynewichorum* in the presence of maxillary and dentary teeth that are distinctly labiolingually wider than mesiodistally long at the base of the crown (character-state 87(2); figure 3*a,b*) and the distinctive mode of tooth replacement with gaps in the tooth row. In addition, the referred partial dentary SMNS 97029 has an edentulous symphysis (character-state 229(1); figure 3*c*), which resembles those of *Trilophosaurus buettneri* [13] and *T. jacobsi* [14] in its anteroposterior extension and differs from the mandibular symphyses of any procolophonid known to date.

The maxillary teeth of *Rutiotomodon tytthos* differ from all other known trilophosaurids except *Trilophosaurus phasmalophos* in the presence of two cusps, the lingual one of which is much smaller than the labial one. The two cusps are subequal in size in *T. phasmalophos* [31]. Teeth of *Trilophosaurus* have three (in *T. buettneri*, *T. dornorum*, and *T. jacobsi*) or two (in *T. phasmalophos*) large, labiolingually aligned cusps, which are linked by transverse occlusal ridges [10,27,31]. *Tricuspisaurus thomasi*, *Variodens inopinatus*, and an indeterminate Middle Triassic trilophosaurid from Nova Scotia each have teeth with three labiolingually positioned cusps [20,33]. By contrast, *Teraterpeton hrynewichorum* has more bulbous maxillary and dentary teeth with two mesiodistally aligned cusps [32].

**Figure 4.**
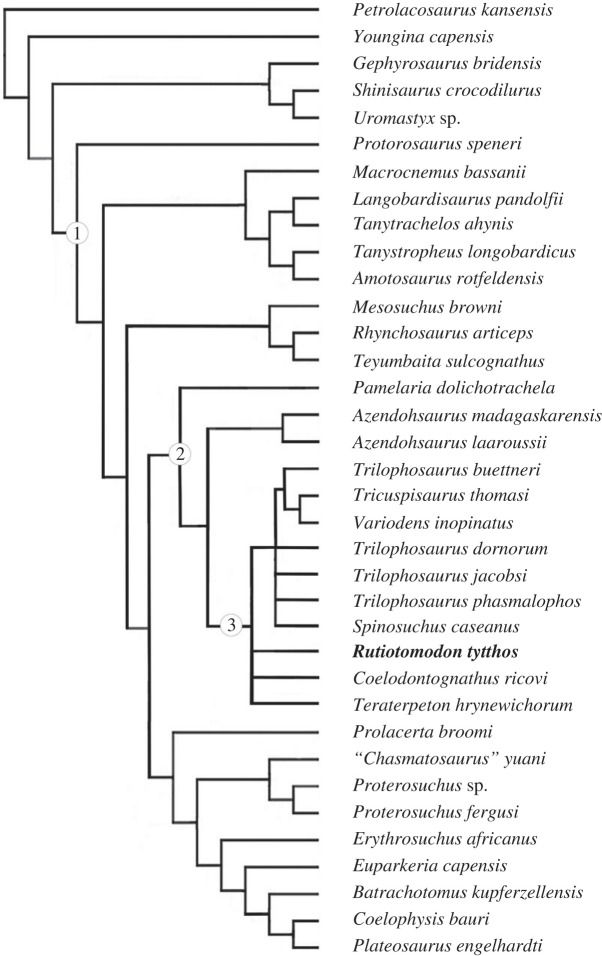
Strict consensus of 40 trees recovered by parsimony analysis of the modified character-taxon matrix from Chambi-Trowell *et al*. [20]. See text for further details. Node (1) represents Archosauromorpha, node (2) Allokotosauria, and node (3) Trilophosauridae. The new taxon *Rutiotomodon tytthos* is highlighted in bold-face type.

**Figure 5.**
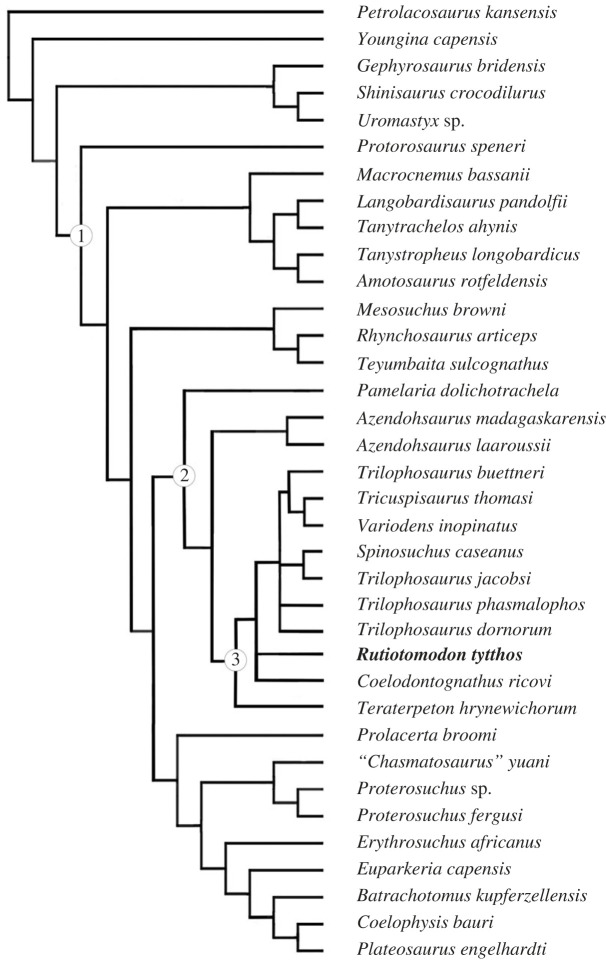
50% majority-rule consensus of 40 trees recovered by parsimony analysis of the modified character-taxon matrix from Chambi-Trowell *et al*. [20]. See text for further details. Node (1) represents Archosauromorpha, node (2) Allokotosauria, and node (3) Trilophosauridae. The new taxon *Rutiotomodon tytthos* is highlighted in bold-face type.

In order to assess the phylogenetic position of *Rutiotomodon tytthos*, we performed a parsimony analysis using the heuristic search option in PAUP* 4.0a [36]. It recovered 40 most parsimonious trees, each with a length of 625 steps, a consistency index of 0.403, a retention index of 0.61, and a rescaled consistency index of 0.258. In the strict consensus tree, there is an unresolved polytomy comprising *Coelodontognathus ricovi*, *Rutiotomodon tytthos*, *Teraterpeton hrynewichorum*, and a clade comprising all other trilophosaurids (figure 4). The 50% majority consensus tree (figure 5) places *Teraterpeton hrynewichorum* as the earliest-diverging taxon and includes two subclades, one with *Trilophosaurus buettneri* and the British trilophosaurids, supported by two unambiguous synapomorphies (character-states 223(2) and 226(1)), and a second with *Trilophosaurus jacobsi* and *Spinosuchus caseanus* (which might be synonymous, as previously suggested [23]). Coding the maxilla and dentary as separate operational taxonomic units (OTUs) did not change these results. The lack of clear resolution among Trilophosauridae can be related to the still limited skeletal remains available for trilophosaurids except for *Trilophosaurus buettneri*, *T. jacobsi*, and *Teraterpeton hrynewichorum*.

*Trilophosaurus buettneri* was originally classified as a procolophonid parareptile [17,18] because, at that time, procolophonids were the only known reptiles with labiolingually wide teeth. Later, *Tricuspisaurus thomasi* was also placed in Procolophonidae [39], but this referral was rejected because *Tricuspisaurus* has protothecodont rather than acrodont tooth implantation [40]. However, this is not a compelling argument because both types of tooth implantation occur together in the procolophonid *Scoloparia glyphanodon* from the Upper Triassic Evangeline Member of the Wolfville Formation in Nova Scotia [41].

Trilophosaurid stem-archosaurs were long known only from the Late Triassic of the United States and Great Britain. However, recent time-calibrated phylogenies of Archosauromorpha have predicted a much longer temporal range of this clade, extending back into the Permian [6,22,42]. The isolated tooth crown from the Economy Member of the Wolfville Formation in Nova Scotia, Canada provided the first evidence of a Middle Triassic trilophosaurid [33]. It forms a flattened platform bearing three labiolingually arranged cusps (character-state 83(1)), the central one of which is the smallest. The Economy Member is considered Middle Triassic (Anisian or Ladinian) in age based on its tetrapod assemblage, which differs considerably in taxonomic composition from that of the overlying, Carnian-age Evangeline Member of the Wolfville Formation [32]. In connection with the present study, we reviewed the affinities of several other taxa of small-bodied reptiles previously referred to Procolophonidae.

Isolated tooth-bearing jaws and teeth from the Lower Triassic (Olenekian) Lipovskaya Svita (Yarenskian Gorizont) of the Volgograd oblast (administrative region) of southwestern Russia were initially assigned to a new procolophonid genus *Coelodontognathus* with two species, *C. ricovi* (type-species) and *C. donensis* [43]. Subsequently, *Coelodontognathus* spp. was referred to Trilophosauridae without further discussion [4].

**Figure 6.**
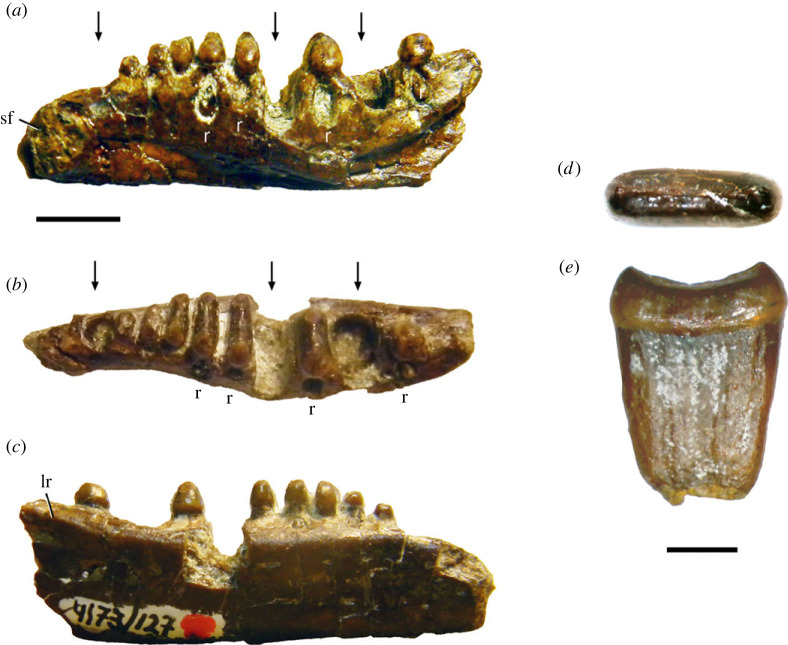
Specimens of *Coelodontognathus ricovi*. (*a–c*) right dentary (PIN 4137/127, holotype) in lingual (*a*), occlusal (*b*), and labial (*c*) views. Arrows in (*a,b*) point to empty alveoli. (*d,e*) referred tooth (PIN 4137/128) in occlusal (*d*) and mesial or distal (*e*) views. Digitally edited from photographs provided by Adam C. Pritchard. Abbreviations: lr, labial (lateral) ridge on dentary; r, crypts with replacement teeth; sf, symphyseal facet of dentary. Scale bars equal 5 mm (*a–c*) and 2 mm (*d,e*).

The holotype of *Coelodontognathus ricovi* is a well-preserved, nearly complete right dentary with teeth (originally SGU 104/3101, now PIN 4137/127; figure 6*a–c*) from the Lower Triassic (Olenekian) Lipovskaya Svita (Yarenskian Gorizont) of the Donskaya Luka locality in the Volgograd oblast [43]. It preserves 10 closely spaced tooth positions. Alveoli 1, 7 and 9 do not contain teeth. Tooth positions 1 and 9 are occupied by empty alveoli, and a deep gap in the tooth row at position 7 was formed by erosion of the labial and lingual alveolar walls. Similar gaps are present in *Rutiotomodon tytthos*, *Trilophosaurus* spp. [13,15,16] and *Tricuspisaurus thomasi* [20] and are the result of tooth replacement, not preservational or artificial damage [16]. Crowns of replacement teeth are visible through foramina lingual to the functional teeth 5, 6, 8 and 10. The crowns of teeth 3 to 6 and 8 are mesiodistally short but labiolingually wide, each with a low labial and lingual cusp linked by a ridge. The mesial and distal margins of the tooth crown are nearly straight transversely whereas the labial and lingual margins are distinctly convex. A referred isolated tooth (originally SGU 104/3102, now PIN 4173/128; figure 6*d,e*) shares this crown configuration. The crowns of teeth 5 and 6 in the holotype have the greatest labiolingual width, similar to the condition in *Trilophosaurus buettneri* [15]. Those of teeth 2 and 10 are more rounded in occlusal outline and less wide labiolingually. As in *Trilophosaurus buettneri* and *T. jacobsi*, the teeth are slightly raised above the alveolar margin. The posteriormost tooth is more dorsally positioned than the penultimate one, similar to the condition in *Trilophosaurus buettneri* [13]. An edentulous region with the width of one tooth position separates the symphyseal facet from the anterior end of the tooth row, unlike the symphyseal ‘beak' in *Trilophosaurus buettneri* and *T. jacobsi* [15] and in *Rutiotomodon tytthos*. The symphyseal facet lacks the ridging present in *Trilophosaurus buettneri*, which has strongly interdigitated symphyseal sutures [13,16]. The relative proportions of the expanded tooth crowns and the mode of tooth replacement closely resemble those in *Trilophosaurus* spp. [16] and are unlike those in known procolophonids [20]. Our phylogenetic analysis found *Coelodontognathus ricovi* among Trilophosauridae [4] more derived than *Teraterpeton hrynewichorum*, as the stratigraphically oldest member of this clade known to date. The robust dentary of *Coelodontognathus ricovi* bears a prominent lateral ridge that extends anteriorly parallel and ventral to the tooth row and resembles the ridges in *Trilophosaurus buettneri* (e.g. TxVP 31025-125) and *Tricuspisaurus thomasi* [20]. This feature is absent in known procolophonids [20]. Posteriorly, the dorsal margin of the dentary rises, forming a coronoid process. The ventral margin of the dentary is narrow transversely, but the body of the tooth-bearing ramus becomes wider labiolingually more dorsally.

A second species of *Coelodontognathus* from the same horizon and locality, *C. donensis*, is based on a right dentary (originally SGU 104/3103, now PIN 4173/129) with a tooth row comprising one partial and eight complete teeth [43]. Its anterior two tooth crowns are rounded in occlusal view whereas teeth 3–7 have labiolingually wide but mesiodistally narrow, bicuspid crowns. *Coelodontognathus donensis* is possibly also a trilophosaurid, but additional information is needed to determine its phylogenetic relationships. *Doniceps lipovensis*, based on a right premaxilla (apparently now lost) from the Lipovskaya Svita of the Donskaya Luka locality [44], may also be referable to Trilophosauridae. Posteriorly, the premaxilla bears three teeth, the second and third of which appear to have labiolingually wide tooth crowns. Its elongate anterior region is edentulous.

*Xenodiphyodon petraios*, from the Upper Triassic (Carnian) Vinita Formation of Virginia, is known from a partial right dentary with teeth (USNM PAL448631) and was originally referred to Procolophonidae based on the assumption that *Tricuspisaurus thomasi* is a procolophonid [45]. Later, it was suggested that this taxon is referable to Trilophosauridae [24]. The dorsoventrally deep but labiolingually narrow dentary of *Xenodiphyodon petraios* is incomplete at its anterior and posterior ends. Its tooth row has at least six mesiodistally aligned, labiolingually narrow teeth with apical ridges anteriorly, followed posteriorly by at least three molariform teeth with labiolingually wide but mesiodistally narrow crowns that each bear three low cusps. The central cusp is the largest. The low height of the cusps is possibly the result of wear or abrasion. The cusps are not connected by ridges. The cusp configuration in *Xenodiphyodon petraios* is most similar to that on the labiolingually wide teeth of *Tricuspisaurus thomasi* [20] and differs from those in known procolophonids. However, the shape of the dentary, especially its transversely narrow tooth-bearing ramus, and the labiolingually narrow anterior teeth differ from the corresponding features in undisputed trilophosaurids, and thus referral to that clade must remain tentative at present.

With the additions of *Coelodontognathus ricovi* from Olenekian and *Rutiotomodon tytthos* from the Ladinian, the known fossil record of Trilophosauridae now spans nearly the entire Triassic Period, from the Olenekian to the Rhaetian. The small body sizes of *Tricuspisaurus thomasi* (1 m or less total length) and *Variodens inopinatus* (*ca* 0.5 m) have been plausibly interpreted as examples of insular dwarfism [20]. However, recognition of the similarly small-bodied *Rutiotomodon tytthos* (less than 0.5 m estimated total length) and *Coelodontognathus ricovi* (*ca* 0.5 m) suggests an alternative hypothesis that the large body size of *Trilophosaurus buettneri* (with an estimated total length of up to 2.5 m [13]) and *T. jacobsi* possibly represents a derived condition for these taxa.

## 5. Palaeobiological inferences

The labiolingually wide teeth of *Rutiotomodon tytthos* and other trilophosaurids resemble those of the Late Cretaceous (Maastrichtian) polyglyphanodontian lizard *Polyglyphanodon sternbergi* [46]. However, the more posterior teeth of the latter species lack cusps and instead have a labiolingually extending occlusal ridge bearing tiny, irregular serrations [47]. Furthermore, the long axes of its tooth crowns extend mesiolabially rather than labiolingually relative to the sagittal plane. In both *Polyglyphanodon sternbergi* and trilophosaurids, the upper and lower teeth interdigitated as the jaws closed and there was no actual tooth-to-tooth occlusion. Presumably, this dental configuration facilitated limited oral processing of plant material by cutting and tearing [47,48]. The anterior ‘beaks' in *Trilophosaurus buettneri*, *T. jacobsi*, and *Rutiotomodon tytthos* presumably served for cropping vegetation.

*Trilophosaurus buettneri* and *Polyglyphanodon sternbergi* have long been considered herbivores [46,47]. Both were large-bodied representatives of their respective clades. Based on the development of its adductor jaw muscles (as inferred from the temporal region of the cranium), *Trilophosaurus buettneri* could generate considerable bite forces, which would have facilitated some oral processing of tough, high-fibre plant fodder. By analogy with extant lizards, the Late Triassic trilophosaurids from Great Britain [20] and *Rutiotomodon tytthos* were small-bodied reptiles that probably could not sustain their metabolic rates on exclusively herbivorous diets [49,50]. Thus, these taxa were presumably omnivores.

*Trilophosaurus buettneri* has several postcranial features, especially the distinctly curved, mediolaterally narrow ungual phalanges, that resemble those of the predominantly tree-dwelling extant lizard *Iguana iguana* and are consistent with a possibly arboreal mode of life [13,15]. Its unguals clearly represent the scansorial type of claws [51]. Small-bodied trilophosaurids such as *Rutiotomodon tytthos* would not necessarily have had anatomical specializations for arboreality. Much like present-day small-bodied lizards such as *Sceloporus occidentalis* [52], they presumably could have moved with ease on any uneven substrates including tree trunks.

The Vellberg fossil *Lagerstätte* preserves the remains of lacustrine vertebrates along with those of terrestrial tetrapods that apparently lived along or near the shoreline of a lake [34]. Occasional finds of carbonaceous mud clasts with attached plant remains have been interpreted as evidence for a relatively near, vegetated shore. However, skeletal remains of putative omnivores and herbivores are uncommon [53], and the absence of undisputed plant consumers such as kannemeyeriiform dicynodonts (such as *Elephantosaurus jachimovitschi* from the correlative Bukobay Gorizont of the southern Urals region of Russia [54]) in the Erfurt Formation suggests low-density vegetation dominated by the horsetail *Equisetites* that may have been mainly restricted to settings near bodies of water.

During the Triassic Period, procolophonids and trilophosaurids were mostly small-sized omnivores and herbivores with dentitions suitable for limited oral processing of food. Procolophonids attained a worldwide distribution [5], whereas trilophosaurids are currently known only from Laurasia. Both clades disappeared near the end of the Triassic, and their ecological roles were subsequently filled by lepidosaurs such as the opisthodontian sphenodontians, which first appeared in the fossil record during the Late Triassic [9,10].

## Data Availability

The data are provided in electronic supplementary material [55].
